# Analysis of ‘One in a Million’ primary care consultation conversations using natural language processing

**DOI:** 10.1136/bmjhci-2022-100659

**Published:** 2023-04-28

**Authors:** Yvette Pyne, Yik Ming Wong, Haishuo Fang, Edwin Simpson

**Affiliations:** 1 Bristol Medical School, University of Bristol Centre for Academic Primary Care, Bristol, UK; 2 Intelligent Systems Labs, University of Bristol, Bristol, UK

**Keywords:** Documentation, Electronic Health Records, Machine Learning, Primary Health Care, Artificial intelligence

## Abstract

**Background:**

Modern patient electronic health records form a core part of primary care; they contain both clinical codes and free text entered by the clinician. Natural language processing (NLP) could be employed to generate these records through ‘listening’ to a consultation conversation.

**Objectives:**

This study develops and assesses several text classifiers for identifying clinical codes for primary care consultations based on the doctor–patient conversation. We evaluate the possibility of training classifiers using medical code descriptions, and the benefits of processing transcribed speech from patients as well as doctors. The study also highlights steps for improving future classifiers.

**Methods:**

Using verbatim transcripts of 239 primary care consultation conversations (the ‘One in a Million’ dataset) and novel additional datasets for distant supervision, we trained NLP classifiers (naïve Bayes, support vector machine, nearest centroid, a conventional BERT classifier and few-shot BERT approaches) to identify the International Classification of Primary Care-2 clinical codes associated with each consultation.

**Results:**

Of all models tested, a fine-tuned BERT classifier was the best performer. Distant supervision improved the model’s performance (F1 score over 16 classes) from 0.45 with conventional supervision with 191 labelled transcripts to 0.51. Incorporating patients’ speech in addition to clinician’s speech increased the BERT classifier’s performance from 0.45 to 0.55 F1 (p=0.01, paired bootstrap test).

**Conclusions:**

Our findings demonstrate that NLP classifiers can be trained to identify clinical area(s) being discussed in a primary care consultation from audio transcriptions; this could represent an important step towards a smart digital assistant in the consultation room.

**WHAT IS ALREADY KNOWN ON THIS TOPIC**

- Natural language processing (NLP) has the potential to revolutionise clinical specialties that rely on free text such as primary care which extensively uses electronic health records.
- Existing NLP tools are focused on classifying free text created by health professionals or generating free text from predefined clinical data.
- The creation of a tool to classify a clinical consultation based on the conversation that occurs in it could have a significant positive effect on clinician workload and could form part of the tools used in an ‘augmented consultation’.

**WHAT THIS STUDY ADDS**

- This study is the first to analyse and classify primary care consultations from the conversations that took place between doctors and patients.
- This study develops and assesses the efficacy of several NLP classifiers, including recent pretrained deep neural networks, for classifying verbatim medical conversation transcripts, which use very different language to clinical notes, and for which extremely limited training data are available.
- This study identifies limitations of the existing healthcare datasets and tools containing primary care free text and makes recommendations for further avenues of research and appropriate data sources.

**HOW THIS STUDY MIGHT AFFECT RESEARCH, PRACTICE OR POLICY**

- This study highlights the importance of building datasets of clinical conversations and other healthcare-based natural language sources for use in clinical research.
- This study suggests several further research topics combining the fields of clinical primary care and machine learning.

## Introduction

Technology is becoming increasingly pervasive in primary care^1^ and a significant proportion of a clinician’s day is spent interacting with the patient electronic health record (EHR). EHRs are a form of ‘handover’, either to another health professional, or to the same clinician when they meet the patient again; the records also provide key evidence in legal cases and are used for performance targets (such as the UK National Health Service Quality and Outcomes Framework) and billing (in the USA). EHRs incorporate free text and clinical codes such as SNOMED-CT, ICD (International Classification of Diseases) or Read codes. Historically, EHRs have been for clinicians only, but incoming UK legislation will open these records to be viewed by patients as well. For all these reasons, it is vital that clinical notes and their associated codes are accurate and complete.

Modern EHR systems used in UK primary care (such as EMIS, SystmOne and Vision) can also direct clinicians to clinically relevant local or national guidelines if the clinician enters an appropriate clinical code. However, clinical codes are often associated with the diagnosis rather than the presenting complaint so may only be entered at the conclusion of the consultation or even after the patient has left. Writing EHR notes or entering clinical codes during a consultation can be disruptive as the clinician has to focus on data capture rather than the patient.^2 3^ Motivated by this, we investigated the first steps towards a natural language processing (NLP)^4^ application that can ‘listen’ to a conversation between general practitioner (GP) and patient and automatically recommend clinical codes.

NLP has previously been applied to healthcare in a wide range of applications; for example, to process and analyse patient feedback,^5^ identify risk factors,^6^ symptoms and treatments,^7^ or suspected disease^8^ from clinical notes, or even to generate notes automatically from structured hospital data.^9^ The technology to transcribe speech to text already exists in tools such as ‘Otter.ai’,^10^ which could enable text processing of clinical conversations. However, the systematic evaluation of the use of NLP for interpreting conversations between clinicians and patients is lacking.^11–14^

We treated the task of assigning clinical codes to transcripts as text classification, which can be addressed using supervised learning. However, training data are in short supply, and recent NLP approaches based on deep learning are data hungry. This research assessed a series of text classifiers trained with small datasets to identify clinical codes associated with real-life GP–patient consultations. Our objectives were to evaluate: (1) the performance of different kinds of text classifiers; (2) the effect of training classifiers using existing medical code descriptions rather than example consultations; (3) the contribution of patients’ speech to correct classifications in addition to the clinician’s speech and (4) opportunities for improving the classifiers in future.

## Methods

### Data sources

#### ‘One in a Million’ dataset

The ‘One in a Million’ (OIAM) dataset^15^ contains 300 video and audio recordings and verbatim transcripts of real clinical consultations conducted in 12 GP practices around Bristol in English with adult patients with permission in place for reuse. These consultations are associated with one or more International Classification of Primary Care (ICPC-2) clinical problem codes assigned by human coders. Both anonymised transcripts and ICPC-2 codes were available for 239 consultations.^16^ A fictional but representative part of a consultation transcript is shown in online supplemental appendix A.

10.1136/bmjhci-2022-100659.supp1 Supplementary data

#### ICPC-2: ICPC-2 code descriptions

This is a primary care focused set of approximately 1300 low-level codes related to clinical problems that are grouped into 17 high level chapters or codes associated with clinical problem areas such as ‘urinary’ or ‘circulatory’.^17^ The ICPC-2e-V.7.0 comma separate values file^18^ was used to create a data dictionary of high-level codes associated with relevant words for that group of conditions.

#### National Institute for Health and Care Clinical Knowledge Summaries

We created a National Institute for Health and Care Clinical Knowledge Summaries (NICE CKS) ‘Health Topics’ dataset using the ‘Web Scraper.io’ Google Chrome extension on 29 July 2021 from the publicly available web resource covering over 370 clinical topics.^19^ For each health topic, we considered text from sections: ‘Causes’, ‘Definition’, ‘Diagnosis’, ‘Clinical features’, ‘History’, ‘Presentation’, ‘Signs and symptoms’ and ‘When to suspect’. The clinical author mapped each NICE CKS topic to one or more ICPC-2 codes (see online supplemental appendix B: ICPC-2 codes and consultations). Then, for each ICPC-2 code, all the related CKS health topics were concatenated into a single document corresponding to that ICPC-2 code. While the ICPC-2 descriptions contain lists of relevant keywords, CKS health topics contain complete sentences that may convey additional information such as descriptions of symptoms.

### Training the NLP classifiers

We initially used the OIAM dataset to train and test a series of classifiers using standard supervised learning (objective (1)). We held out a stratified sample of 20% (48 transcripts) of OIAM as a test set, using the remainder (191 transcripts) for training. Hyperparameter tuning was performed using fivefold cross-validation on the training split (see online supplemental appendix D).

Supervised learning requires a training dataset containing sufficiently representative examples for each class label, yet our training set contains only a small number of example consultations per code. We, therefore, introduced a second approach, ‘distant supervision’, that used the ICPC-2 code descriptions and NICE CKS datasets as training examples and tested the classifiers on the OIAM dataset (objective 2). We also tested excluding the ‘A: General’ classification as it includes a wide spectrum of clinical conditions from ‘pain general/multiple sites’ to ‘viral disease other’, and thus assigning the code was unlikely to aid GPs and may confuse the classifiers. Finally, we analysed distant supervision performance considering only the GP’s half of the conversation to determine whether transcribing patient’s speech is beneficial (objective 3).

To assess classifier performance, we used the macroaverage precision (equivalent to positive predicted value; the fraction of labels assigned by the classifier that were correct), recall (also called ‘sensitivity’; the fraction of true labels predicted by the classifier) and F1 score (the harmonic mean of precision and recall).

As a baseline, we assigned labels at random, allowing multiple labels per transcript. We tested shallow, data-efficient classifiers: naïve Bayes (NB), as a linear classifier; support vector machine (SVM) with RBF kernel, a non-linear classifier that performs well with high dimensional feature vectors, such as those used to represent text (see below); and nearest centroid with Euclidean distance as a lightweight clustering-based classifier. While there are many other alternatives, the chosen methods represent broad types of classifier and allowed us to determine the suitability of classifiers with increasing complexity (part of objective (1)). The NB and SVM classifiers were run in ‘multilabel’ and ‘multiclass’ classification modes:

- Multilabel: for each possible ICPC-2 code, we train a binary classifier to assign either ‘yes’ or ‘no’ per consultation, so that more than one code can be assigned to the consultation.
- Multiclass: we train one classifier to assign the single most likely ICPC-2 code to the consultation. In training, we select the first code for each consultation, with codes sorted alphabetically.

In both modes, classifiers were evaluated on the complete test dataset using the same metrics. For consultations with more than one ICPC-2 code, the correct set of labels must be predicted to achieve perfect recall. As there are 110 consultations with more than one label, this puts a ceiling on the recall of the multiclass approach. However, the training data are more balanced, which may lead to better recall than the multilabel setup, where the training data for each binary classifier contains only a small minority of positive examples. Precision could also be higher as the multiclass mode directly compares classes that are easy to confuse.

For the shallow classifiers, we removed stopwords from the consultation transcripts before processing them. Considering the choice of stopwords as part of objective (1), we tested 3 sets: 318 ‘English’ stopwords (from sklearn’s default ENGLISH_STOP_WORDS); 203 ‘medical’ stopwords^20^ and 61 ‘custom’ stopwords (see online supplemental appendix C: custom stopword dictionary). We encoded each transcript, ICPC-2 code description and CKS health topic as a feature vector containing the counts of the 5000 most frequent unigrams (individual words) and bigrams (consecutive pairs of words).

We also trialled recent deep learning classifiers that leverage a pretrained transformer, PubMedBERT,^21^ a variant of BERT^22^ that was pretrained on biomedical text (objective (1)). PubMedBERT encodes text into dense vector representations that take word order into account and include medical terms not present in our training examples. We tested a ‘conventional BERT’ classifier, in which we fine-tuned a classification head on top of PubMedBERT (multiclass mode). For distant supervision, we compared this to two BERT setups designed for training with very few examples: using next sentence prediction (NSP) to compare the text to a prompt containing the name of each class (multilabel mode); and using masked language modelling (MLM) to predict the category name by filling in the blank word in a prompt (multiclass)^23^; both used ‘this is a problem of ___’ as a prompt. We hypothesised that the BERT approaches would outperform shallow classifiers thanks to their pretrained language representations, and that MLM would perform best as it reuses the pretraining task, so does not need to learn new classifier layers from scratch. Since BERT has a length limit of 512 tokens, transcripts and CKS topics were broken into multiple documents consisting of complete sentences. For training, all chunks were assigned the corresponding ICPC-2 training label. For prediction, we took the union of labels predicted for each of the chunks.

## Results

**Figure 1:**
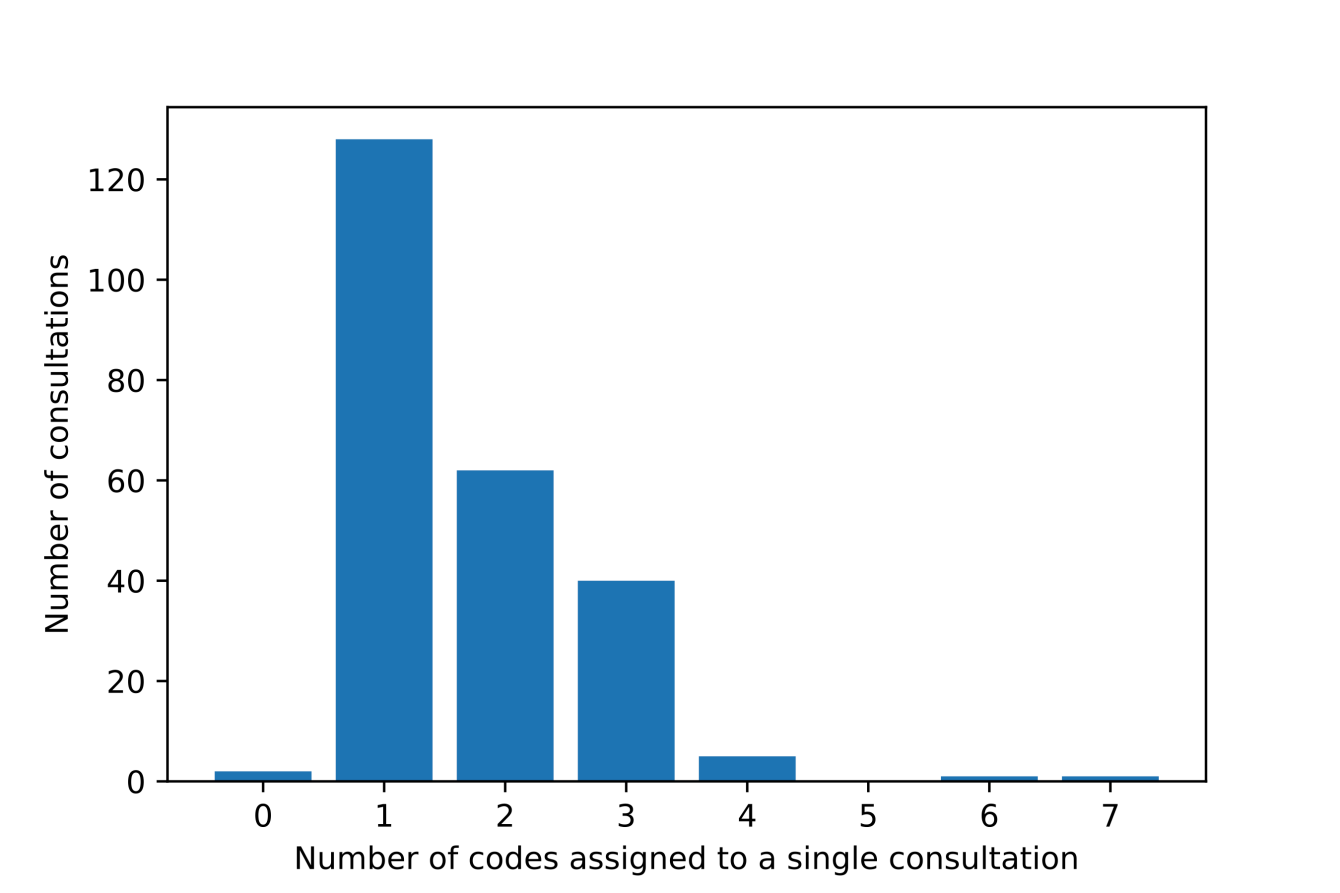
Distribution of consultations with multiple labels.

The consultation and patient demographics for the OIAM dataset are given in table 1, and the number of transcripts with multiple labels is shown in figure 1.

**Table 1:** Details of the OIAM dataset used in this work, with patient information for the complete dataset

ICPC-2 code	No of transcripts	%
A: General	14	5.9
B: Blood, blood forming	8	3.3
D: Digestive	44	18.4
F: Eye	5	2.1
H: Ear	11	4.6
K: Circulatory	32	13.4
L: Musculoskeletal	65	27.2
N: Neurological	20	8.4
P: Psychological	50	20.9
R: Respiratory	37	15.5
S: Skin	32	13.4
T: Metabolic, endocrine, nutritional	24	10.0
U: Urinary	18	7.5
W: Pregnancy, family planning	11	4.6
X: Female genital	14	5.9
Y: Male genital	7	2.9
Total ICPC-2 code labels	392	164
Total unique consultations	239	100
No of ICPC-2 codes assigned to a consultation (see figure 1)		
0	2	1
1	128	53
2	62	26
3	40	17
4+	8	3
Duration (minutes)		
<5	13	5.4
5–10	79	33.1
10–15	82	34.3
15–20	52	21.8
20–35	13	5.4
Dataset statistics below are for the original patient sample of N=334.^16^ This information was not available to compute for the N=239 subset in our experiments	No of patients	**%**
Sex		
Female	212	63.5
Male	122	36.5
Age		
18–34	91	27.2
35–54	94	28.1
55–74	99	29.6
≥75	36	10.8
Not reported	14	4.2
Ethnic group		
White	291	87.1
Other	43	12.9
IMD (Indices of Multiple Deprivation) quintile		
1st (least deprived)	106	31.7
2nd	54	16.2
3rd	35	10.5
4th	53	15.9
5th (most deprived)	84	25.1
Data unavailable	2	0.6

ICPC-2, International Classification of Primary Care-2; OIAM, One in a Million.

### Objective (1): types of NLP classifiers

Table 2 shows the results for classifiers trained on OIAM transcript texts, with best performances highlighted in bold. As the held-out test set is small, we include the results of fivefold cross-validation over the larger training set. Nearest centroid is the best shallow classifier. Multiclass NB clearly outperforms SVM, while BERT provides substantial improvements all round. Compared with multilabel mode, multiclass classifiers have higher precision. However, recall and F1 are lower for multiclass SVM, while they are higher for multiclass NB, despite being unable to assign multiple codes to a single transcript. The baseline slightly outperforms multilabel NB on the test set and is competitive with some other shallow methods.

**Table 2:** Performance with standard supervised learning, 95% CIs shown in parentheses

	Validation			Train	Test		
**Model**	**Precision**	**Recall**	**F1**	**F1**	**Precision**	**Recall**	**F1**
Random baseline	0.499	0.104	0.161	0.161	0.501	0.102	0.158
Conventional supervision							
Naïve Bayes (multilabel)	0.284 (0.237 to 0.325)	0.140 (0.135 to 0.192)	0.175 (0.161 to 0.222)	**0.999**	0.234 (0.169 to 0.276)	0.113 (0.087 to 0.158)	0.139 (0.106 to 0.185)
Naïve Bayes (multiclass)	0.372 (0.298 to 0.399)	0.327 (0.314 to 0.398)	0.300 (0.266 to 0.342)	0.696	0.178 (0.146 to 0.232)	0.238 (0.213 to 0.294)	0.181 (0.154 to 0.226)
SVM (multilabel)	0.107 (0.112 to 0.132)	**1.000** (1.000 to 1.000)	0.184 (0.192 to 0.223)	0.181	0.102 (0.095 to 0.124)	1.000 (1.000 to 1.000)	0.177 (0.166 to 0.211)
SVM (multiclass)	0.200 (0.171 to 0.244)	0.159 (0.157 to 0.211)	0.154 (0.142 to 0.196)	0.696	0.217 (0.145 to 0.263)	0.169 (0.14 to 0.227)	0.164 (0.129 to 0.213)
Nearest centroid (multiclass)	0.349 (0.297 to 0.395)	0.270 (0.254 to 0.327)	0.278 (0.247 to 0.325)	0.694	0.307 (0.18 to 0.355)	0.205 (0.15 to 0.276)	0.219 (0.151 to 0.278)
BERT conventional (multiclass)	**0.467** (0.434 to 0.549)	0.577 (0.546 to 0.654)	**0.480** (0.447 to 0.550)	0.696	**0.484** (0.414 to 0.575)	**0.509** (0.434 to 0.610)	**0.452** (0.390 to 0.525)
Distant supervision							
Naïve Bayes (multilabel), ICPC-2	0.626 (0.515 to 0.687)	0.234 (0.196 to 0.278)	0.323 (0.268 to 0.362)	0.979	0.590 (0.427 to 0.656)	0.285 (0.206 to 0.384)	0.378 (0.274 to 0.456)
Naïve Bayes (multiclass), ICPC-2	0.516 (0.466 to 0.569)	0.590 (0.541 to 0.639)	0.512 (0.462 to 0.549)	1.00	0.511 (0.412 to 0.611)	0.524 (0.449 to 0.628)	0.481 (0.404 to 0.567)
Nearest centroid, ICPC-2	**0.718** (0.565 to 0.765)	0.416 (0.373 to 0.463)	0.444 (0.384 to 0.489)	1.00	0.520 (0.400 to 0.615)	0.362 (0.298 to 0.448)	0.386 (0.303 to 0.467)
Conventional BERT, CKS	0.603 (0.553 to 0.653)	0.584 (0.53 to 0.64)	0.550 (0.494 to 0.593)	0.927	**0.551** (0.477 to 0.649)	0.562 (0.483 to 0.691)	**0.508** (0.429 to 0.594)
BERT NSP, CKS	0.364 (0.333 to 0.394)	**0.816** (0.767 to 0.865)	0.462 (0.424 to 0.488)	0.291	0.257 (0.215 to 0.331)	**0.598** (0.525 to 0.711)	0.306 (0.257 to 0.371)
BERT MLM, CKS	0.600 (0.547 to 0.64)	0.615 (0.566 to 0.673)	**0.567** (0.512 to 0.604)	0.792	0.481 (0.409 to 0.574)	0.536 (0.469 to 0.639)	0.467 (0.397 to 0.548)

For conventional supervision, ‘train’ and ‘test’ results are for classifiers trained on the whole 80% training split, and validation was performed using 5-fold cross-validation over the training set. For distant supervision, the OIAM training set was repurposed as a validation set, as it was not used to train the models with this setup.

CKS, Clinical Knowledge Summaries; ICPC-2, International Classification of Primary Care-2; MLM, masked language modelling; NSP, next sentence prediction; OIAM, One in a Million; SVM, support vector machine.

A comparison of F1 scores with different stopwords is shown in table 3, with the best choice for each classifier in bold, corresponding to the results in Table 2. Removing English or medical stopwords is helpful, while removing the words in all three stopword lists is most effective.

**Table 3:** F1 scores for fivefold cross-validation performance on the OIAM training set with different sets of stopwords

Model	No removal	English	Medical	Custom	Medical+custom	English+custom	English+medical +custom
Naïve Bayes (multilabel)	0.157	0.159	0.154	0.143	0.166	0.170	**0.175**
Naïve Bayes (multiclass)	0.225	0.266	0.243	0.228	0.245	0.272	**0.300**
SVM (multilabel)	0.184	0.184	0.184	0.184	0.184	0.184	0.184
SVM (multiclass)	0.141	0.151	0.141	0.142	0.142	0.150	**0.154**
Nearest centroid	0.234	0.256	0.239	0.234	0.247	0.252	**0.278**

OIAM, One in a Million; SVM, support vector machine.

### Objective (2): distant supervision

Table 4 compares F1 scores for different stopword lists with distant supervision. With CKS, the combined list is again most effective, but medical stopword removal is detrimental with ICPC-2 descriptions. Since ICPC-2 descriptions contain keywords rather than prose, any medical stopwords included by the authors of the descriptions may be part of informative key phrases that should not be removed.

**Table 4:** F1 scores for distant supervision performance, evaluated on the OIAM training set, with different sets of stopwords, and training on either CKS topics or ICPC-2 descriptions

Model	No removal	English	Medical	Custom	Medical+custom	English+custom	English+medical+custom
NB (multilabel), ICPC-2	0.139	0.170	0.136	0.253	0.297	**0.323**	0.297
NB (multilabel), CKS	0.096	0.160	0.119	0.126	0.191	0.207	**0.234**
NB (multiclass), ICPC-2	0.324	0.354	0.307	0.461	0.471	**0.512**	0.470
NB (multiclass), CKS	0.245	0.274	0.249	0.275	0.340	0.368	**0.375**
Nearest centroid, ICPC-2	0.312	0.354	0.317	0.432	0.437	**0.445**	0.437
Nearest centroid, CKS	0.326	0.349	0.344	0.349	0.353	0.357	**0.365**

CKS, Clinical Knowledge Summaries; ICPC-2, International Classification of Primary Care-2; NB, Naïve Bayes; OIAM, One in a Million.

Table 5 compares performance on the OIAM training set using distant supervision with the ICPC-2 code descriptions and NICE CKS topics. NB performs best with ICPC-2 supervision, in this case outperforming nearest centroid. BERT does not match the performance of NB multiclass on this small training set and conventional BERT fails to learn at all. BERT variants perform better with CKS than ICPC-2 as PubMedBERT was pretrained to process prose, rather than keywords. Combining both distant supervision sources does not improve performance for any of the methods (table 6).

**Table 5:** F1 scores for different sources of distant supervision, and the effect of removing class A, evaluated on the OIAM training set

Model	ICPC-2	ICPC-2 without A	CKS	CKS without A	ICPC-2 and CKS combined	Combined without A
Naïve Bayes (multilabel)	0.323 (0.268, 0.362)	**0.345** (0.286, 0.389)	0.234 (0.196, 0.262)	0.249 (0.207, 0.285)	0.254 (0.21, 0.287)	0.271 (0.225, 0.308)
Naïve Bayes (multiclass)	**0.512** (0.462, 0.549)	0.508 (0.458, 0.546)	0.375 (0.325, 0.411)	0.391 (0.34, 0.428)	0.378 (0.33, 0.416)	0.385 (0.338, 0.421)
Nearest centroid	0.444 (0.384, 0.489)	0.093 (0.063, 0.12)	0.365 (0.312, 0.401)	0.086 (0.057, 0.107)	0.367 (0.315, 0.403)	0.090 (0.063, 0.113)
BERT conventional	0.057 (0.049, 0.065)	0.027 (0.02, 0.037)	0.550 (0.494, 0.593)	**0.521** (0.459, 0.565)	**0.540** (0.476, 0.576)	**0.545** (0.483, 0.590)
BERT NSP	0.285 (0.232, 0.324)	0.347 (0.309, 0.371)	0.462 (0.424, 0.488)	0.434 (0.392, 0.466)	0.445 (0.402, 0.476)	0.467 (0.425, 0.498)
BERT MLM	0.505 (0.444, 0.544)	0.486 (0.425, 0.528)	**0.567** (0.512, 0.604)	0.497 (0.441, 0.535)	0.532 (0.472, 0.571)	0.475 (0.424, 0.512)

Highest F1 scores in bold.

CKS, Clinical Knowledge Summaries; ICPC-2, International Classification of Primary Care-2; MLM, masked language modelling; NSP, next sentence prediction; OIAM, One in a Million.

**Table 6:** F1 scores when patients’ transcribed speech is excluded

Model	Including GP and patient speech	Only GP speech
Naïve Bayes (multilabel) ICPC-2	0.323 (0.268, 0.362)	**0.372** (0.3, 0.417)
Naïve Bayes (multiclass) ICPC-2	**0.512** (0.462, 0.549)	0.484 (0.429, 0.521)
Nearest centroid ICPC-2	**0.444** (0.384, 0.489)	0.425 (0.361, 0.47)
BERT conventional, CKS	**0.550** (0.494, 0.593)	0.445 (0.384, 0.465)
BERT NSP, CKS	**0.462** (0.424, 0.488)	0.436 (0.398, 0.464)
BERT MLM, CKS	**0.567** (0.512, 0.604)	0.500 (0.434, 0.539)

The classifiers were trained using their most effective distant supervision source and evaluated on the OIAM training set (repurposed as a validation set). Bold indicates best performance in a comparison between including and excluding patients’ speech with the same classifier.

CKS, Clinical Knowledge Summaries; GP, general practitioner; ICPC-2, International Classification of Primary Care-2; MLM, masked language modelling; NSP, next sentence prediction; OIAM, One in a Million.

Table 2 also shows that removing the option of assigning class A causes a collapse in performance with BERT NSP and MLM with ICPC-2 descriptions, and nearest centroid with either supervision source, while NB is improved slightly.

Table 2 shows test set performance with the most successful distant supervision source for each classifier. In comparison with standard supervision, the performance improves substantially for most classifiers, validating the use of external sources for distant supervision.

**Figure 2:**
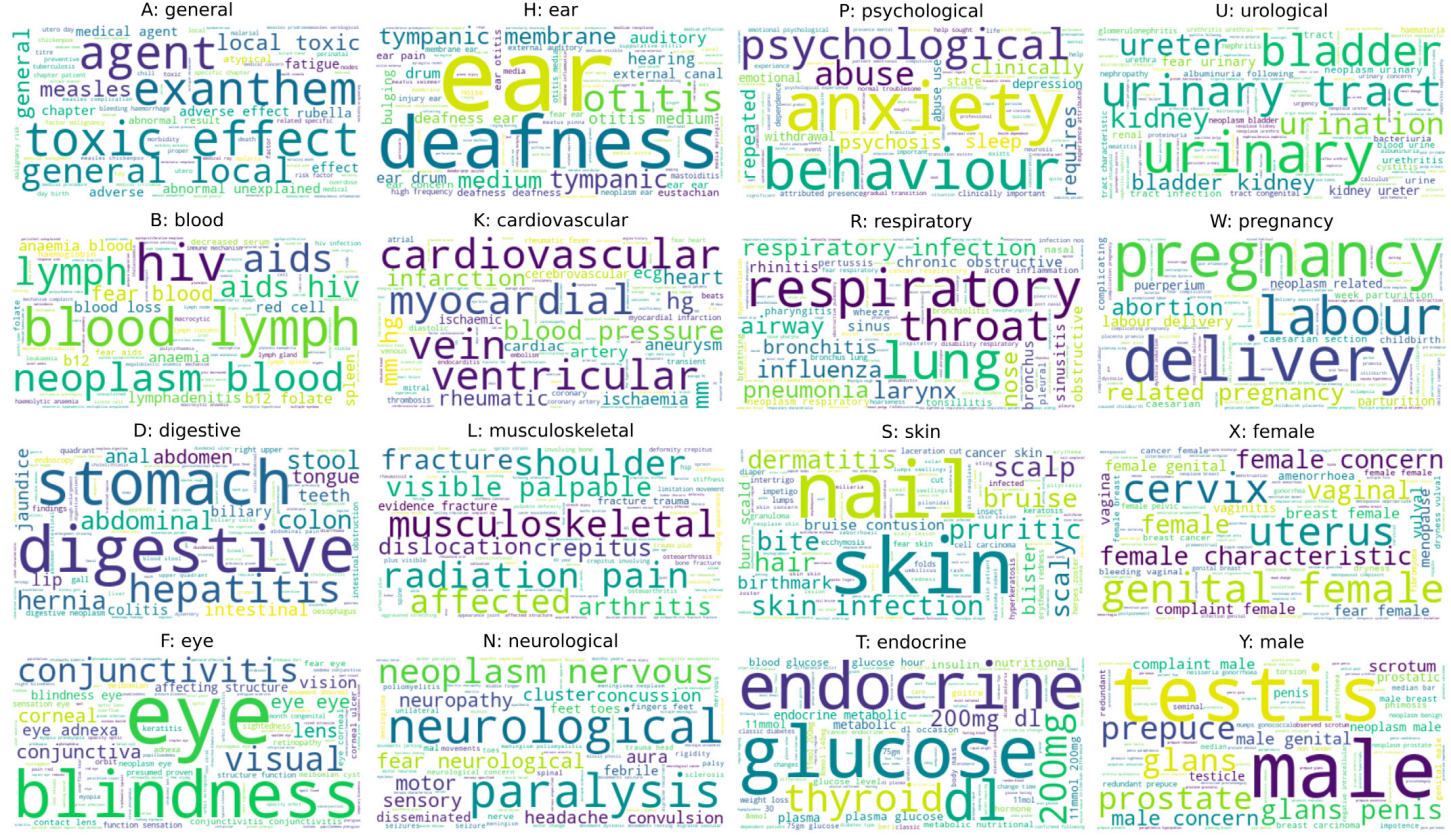
Wordclouds for each ICPC-2 category, with unigrams and bigrams weighted by the probability of the class label given the feature. ICPC-2, International Classification of Primary Care.

The NB model allows direct interpretation of the important features for classification. The wordclouds in figure 2 show the unigrams and bigrams for each class, weighted by the probability of the class given the feature, as learnt by NB (multiclass) from ICPC-2 descriptions. The informative features correspond well with medical terms in each category, but we do not see colloquial terms that may be used in conversation, or expressions longer than two tokens. Therefore, classifiers may benefit from augmenting ICPC-2 descriptions with alternative terms and phrases (objective 4, future improvements).

### Objective (3): contribution of patient speech transcripts

Table 6 shows that using only the GP’s part of the transcript reduces performance of most classifiers, indicating that patients provide useful information that is not contained in the GP’s speech. The CIs only indicate strong evidence of a performance difference for BERT conventional, hence the finding may require investigation with a larger dataset.

## Discussion

We evaluated a range of text classifiers, achieving the highest F1 score on the test set of 0.51 for conventional BERT, with recall at 56% and precision at 55%, substantially better than n-gram-based classifiers (objective 1). This classifier was trained on medical code descriptions, which outperformed standard supervision with a training set of 191 transcripts (those with no missing data such as codes, transcripts or notes) with F1=0.45 (objective 2). When patients’ speech transcripts were excluded, the performance also dropped from F1=0.55 to 0.45 showing that is beneficial to capture the complete conversation (objective 3). Below, we identify specific ways to further improve the classifiers (objective 4).

More work is required to determine whether classifiers with this level of performance could usefully assist clinicians. Our scores are at the lower end of results for comparable multiclass text categorisation tasks,^24^ which achieved between 53% and 86% average accuracy using a RoBERTa classifier with 100 training examples, and substantially lower than BERT for intent classification on dialogue benchmarks,^25^ which achieves almost 93% accuracy with 10 training examples. Future work could, therefore, draw on these related tasks to identify improvements to the classifiers.

NB was competitive with BERT suggesting that unigrams and bigrams provide strong signals about health topics, and that datasets on the scale of OIAM may be insufficient to make full use of deep models. Against our expectations, conventional BERT was marginally the strongest, outperforming BERT MLM on the test set. The BERT models are costly to run (several hours GPU training for all BERT variants vs a few seconds with NB; testing takes in around 100 times longer), although this may not be an issue if training is performed only once before deploying the model. Future work could investigate replacing PubMedBERT with other domain-specific pretrained models (such as BioBERT^26^ and ClinicalBERT^27^). Extremely large language models (LLMs) may also offer improved few-shot learning, although extensive prompt engineering is required and computational costs are huge. These LLMs could potentially generate explanations of their decisions that could bring relevant parts of the conversation to a doctor’s attention.

The multilabel classifiers did less well than the multiclass classifiers, possibly because their training data was highly imbalanced (harming recall) or because multiple labels were assigned in cases where only one of the labels should have been chosen (hurting precision). However, given the complexity and breadth of primary care consultations, any effective classifier would need to be able to suggest multiple medical areas, so multilabel methods must be a focus for future research.

Given the low numbers of examples of some codes (eg, only five consultations were coded as ‘F: eye’), overfitting was an issue for supervised learning, with higher performance on the training set than the validation and test sets. Distant supervision with the NICE CKS Health Topics and ICPC-2 Code descriptions demonstrated clear improvements. The key phrases in the ICPC-2 descriptions are a natural fit for NB: these features are individually informative, which allows linear models such as NB to perform well. The imperfect mapping between CKS topics and ICPC-2 codes may reduce the performance of NB on CKS topics. Improving the mapping would require costly manual editing of the scraped CKS health topics, as some CKS topics lack a one-to-one mapping to an ICPC-2 code. Still, CKS topics produce competitive performance with BERT, which was pretrained with complete sentences, suggesting that the health topics do include useful training signals. Future work could, therefore, investigate ensembles that stack^28^ models trained with different sources of data.

To identify common classifier mistakes, the clinician on the research team reviewed individual consultation transcripts and their human and predicted codes and noted several distinct types of errors. First, shallow classifiers demonstrated simple linguistic errors such as misunderstanding idioms. In one consultation, the GP repeatedly mentioned ‘keeping an eye on it’ and the NB classifier incorrectly coded it as an ophthalmology-related consultation; BERT overcame this by avoiding reliance on isolated words as features.^29^ Second, perusing specific consultations where the NLP classifier appeared to get the coding significantly wrong highlighted errors by the original human labelling team. Third, the ‘A: General’ category was often selected erroneously, as the class is non-specific (precision=0.154 for NB multiclass, trained on ICPC-2 descriptions), although excluding this class often hurt performance. Finally, there were examples where a lack of clinical knowledge caused errors such as the NLP classifier assuming that a consultation discussing someone’s wrist was a musculoskeletal rather than a neurological issue (such as in carpal tunnel syndrome).

Many of these specific types of error relate to limitations of the dataset: its scale, labelling quality and labelling scheme; we consider its small size to be the most significant issue. When scaling up the dataset, further limitations to address include the dataset being only in English and all the consultations taking place in one part of the UK. The current areas where clinical machine learning is excelling are radiology and pathology due to their large and accessible (anonymised) datasets, and the creation of a large, anonymised, free text dataset related to primary care would be hugely valuable for research. The COVID-19 pandemic accelerated the use of online consultations producing potential sources of patient-entered free text (eg, AskMyGP^30^) and recorded audio/video consultations for examination (eg, by FourteenFish^31^). We advocate for routinely incorporating consent to use digitally recorded clinical consultations for research and providing robust anonymisation of them, so that researchers can conduct valuable and translational research in this area.

Further directions for future research include processing the consultations in ‘real-time’ and assigning them to the more fine-grained NICE CKS health topics rather than ICPC-2 codes, which would allow the system to link a doctor automatically to the corresponding health topic guidelines. Performance may also be improved by combining text with other data from electronic medical records.

## Conclusion

This paper offers a promising avenue of research using NLP to extract information from the conversation between a patient and their doctor in a primary care consultation and demonstrates a successful collaboration between clinical and computing disciplines. Previous projects using NLP in a clinical setting have focused on classifying free text created by health professionals (such as radiology reports) or generating free text from codes and defined data (such as investigation results). To our knowledge, this is the first time that the original conversation between a doctor and their patient has been analysed using NLP. Our comparison of text classifiers showed modest gains from deep learning approaches, that the models can be trained using health topics scraped from web pages, and that patients’ speech contains valuable signals for assigning medical codes. We identified potential improvements, including adding colloquial vocabulary to health topic descriptions, increasing the dataset size and domain-specific pretraining of language models. Our ultimate goal would be to provide a smart digital assistant that can create effective consultation notes and suggest questions or guidelines to the clinician^32^; this is likely to require significant advances both in NLP and in our understanding of what makes good clinical notes. While this goal is still a long way off, our work represents one small step towards that reality.

## Data Availability

The ‘One in a Million’ dataset is available for research use following valid ethics approval.ICPC-2 Codes and descriptions are freely downloadable from the web. The NICE CKS Health Topics dataset is freely downloadable from their website using freely available ‘web-scraping’ tools.
