# Supplementary material for: Analysis of ‘One in a Million’ primary care consultation conversations using natural language processing
Source: BMJ Health Care Inform. 2023 Apr 28;30(1):e100659. doi: 10.1136/bmjhci-2022-100659 (PMC10151863; doi:10.1136/bmjhci-2022-100659)
Supplement: Supplementary data [file bmjhci-2022-100659supp001.pdf]

Analysis of "One in a Million" Primary Care Consultation Conversations using Natural Language Processing

Appendix A: Fictional Representative Partial Transcript Example

GP: Hello, I'm Doctor [REDACTED]; thank you for waiting. I'm just going to read out that number you have on that piece of paper if that's okay? 123456 Great, thank you for that, what is troubling you today?

Patient: My headache is carrying on and on doctor. It's been weeks and nothing seems to be making it any better. There were some blood tests done, what happened about them?

GP: Oh, I'm sorry to hear about your headache, let me look on the system for the results.

Patient: Okay.

GP: Oh, that's not loading up at the moment, tell me more about your pain.

Patient: Yes, I need some extra medicine because it's very painful.

GP: Okay are you taking anything at the moment?

Patient: Paracetamol and Ibuprofen It's not helping at all.

GP: You say it's not helping at all?

Patient: No.

GP: Okay, so where are the headaches happening when they come on?

Appendix B: ICPC-2 Codes & Consultations

ICPC-2 Code	CKS Health Topic
A General	Adverse drug reactions, AIDS and HIV infection, Analgesia - mild-to-moderate pain, Angio-oedema and anaphylaxis, Childhood cancers - recognition and referral, Chronic pain, Corticosteroids - oral, DMARDs, Drugs - adverse reactions, End of life care in children, Falls - risk assessment, Faltering growth, Feverish children - management, Feverish children - risk assessment, Glandular fever (infectious mononucleosis), Healthcare-associated infections, Hiccups, Immunizations - childhood, Immunizations - travel, Infectious mononucleosis - Glandular fever, Lyme disease, Malaria, Malaria prophylaxis, Measles, Multimorbidity, Mumps, NSAIDs - prescribing issues, Opioid dependence, Palliative cancer care - pain, Palliative care - general issues, Parvovirus B19 infection, Poisoning or overdose, Roundworm, Rubella, Scarlet fever, Sleep disorders - shift work and jet lag, Tamoxifen - managing adverse effects, Tiredness/fatigue in adults, Travel immunizations
B Blood, blood-forming	Anaemia - B12 and folate deficiency, Anaemia - iron deficiency, Anticoagulation - oral, Antiplatelet treatment, B12 and folate deficiency anaemia, Bruising, Erythrocytosis/polycythaemia, Folate and B12 deficiency anaemia, Gout, Haematological cancers - recognition and referral, HIV infection and AIDS, Hypercholesterolaemia - familial, Iron deficiency anaemia, Multiple myeloma, Neutropenic sepsis, Nosebleeds (epistaxis), Platelets - abnormal counts and cancer, Polycythaemia/erythrocytosis, Sepsis, Sickle cell disease, Sinusitis
D Digestive	Anal fissure, Aphthous ulcer, Appendicitis, Bowel screening, Candida - oral, Coeliac disease, Colic - infantile, Constipation, Constipation in children, Cow's milk allergy in children, Crohn's disease, Dental abscess, Diarrhoea - adult's assessment, Diarrhoea - antibiotic associated, Diarrhoea - prevention and advice for travellers, Diverticular disease, Dyspepsia - pregnancy-associated, Dyspepsia - proven functional, Dyspepsia - proven GORD, Dyspepsia - proven peptic ulcer, Dyspepsia - unidentified cause, Gallstones, Gastroenteritis, Gastrointestinal tract (lower) cancers - recognition and referral, Gastrointestinal tract (upper) cancers - recognition and referral, Gingivitis and periodontitis, GORD in children, Halitosis, Head and neck cancers - recognition and referral, Herpes simplex - oral, Irritable bowel syndrome, Nausea/vomiting in pregnancy, Palliative care - constipation, Palliative care - nausea and vomiting, Palliative care - oral, Periodontitis and gingivitis, Pilonidal sinus disease, Pruritus ani, Teething, Threadworm, Ulcerative colitis, Ulcers - aphthous
F Eye	Age-related macular degeneration, Blepharitis, Cataracts, Chalazion (meibomian cyst), Conjunctivitis - allergic, Conjunctivitis - infective, Corneal superficial injury, Dry eye syndrome, Glaucoma, Herpes simplex - ocular, Hordeola (styes), Macular degeneration - age-related, Meibomian cyst (chalazion), Red eye, Retinal detachment, Squint in children, Styes (hordeola), Uveitis
H Ear	Cholesteatoma, Earwax, Hearing loss in adults, Otitis externa, Otitis media - acute, Otitis media - chronic suppurative, Otitis media with effusion
K Circulatory	Angina, Atrial fibrillation, Cardiac arrest - out of hospital care, Chest pain, Chilblains, Compression stockings, CVD prevention - lipid modification, CVD risk assessment and management, Deep vein thrombosis, DVT prevention for travellers, Giant cell arteritis, Haemorrhoids, Heart failure - chronic, Hypertension, Hypertension in pregnancy, Leg ulcer - venous, Lipid modification - CVD prevention, Lipodermatosclerosis and venous eczema, MI - secondary prevention, Palpitations, Peripheral arterial disease, Raynaud's phenomenon, Superficial vein thrombosis (superficial thrombophlebitis), Varicose veins, Venous eczema and lipodermatosclerosis
L Musculoskeletal	Achilles tendinopathy, Acute childhood limp, Ankylosing spondylitis, Back pain - low (without radiculopathy), Baker's cyst, Bone and soft tissue sarcoma - recognition and referral, Bunions, Bursitis - pre-patellar, Carpal tunnel syndrome, Cervical radiculopathy - neck pain, Chest pain, Childhood limp - acute, Developmental rheumatology in children, Dupuytren's disease, Greater trochanteric pain syndrome, Knee pain - assessment, Leg cramps, Limp (childhood) - acute, Low

	back pain (without radiculopathy), Morton's neuroma, Neck lump, Neck pain - acute torticollis, Neck pain - cervical radiculopathy, Neck pain - non-specific, Neck pain - whiplash injury, Olecranon bursitis, Osgood-Schlatter disease, Osteoarthritis, Osteoporosis - prevention of fragility fractures, Plantar fasciitis, Polymyalgia rheumatica, Pre-patellar bursitis, Restless legs syndrome, Rheumatoid arthritis, Sarcoma (bone and soft tissue) - recognition and referral, Sciatica (lumbar radiculopathy), Shoulder pain, Sprains and strains, Temporomandibular disorders (TMDs), Tennis elbow, Torticollis (acute) - neck pain, Whiplash injury - neck pain
N Neurological	Bacterial meningitis and meningococcal disease, Bell's palsy, Benign paroxysmal positional vertigo, Blackouts, Brain and central nervous system cancers - recognition and referral, Carbon monoxide poisoning, Central nervous system and brain cancers - recognition and referral, Cerebral palsy, Cervical radiculopathy - neck pain, Delirium, Dementia, Epilepsy, Febrile seizure, Head injury, Headache - assessment, Headache - cluster, Headache - medication overuse, Headache - tension-type, Hearing loss in adults, Learning disabilities, Meniere's disease, Meningitis - bacterial meningitis and meningococcal disease, Migraine, Morton's neuroma, Multiple sclerosis, Neuralgia - post-herpetic, Neuropathic pain - drug treatment, Parkinson's disease, Post-herpetic neuralgia, Radiculopathy (cervical) - neck pain, Radiculopathy (lumbar) - sciatica, Sciatica (lumbar radiculopathy), Stroke and TIA, Tinnitus, Trigeminal neuralgia, Vertigo, Vertigo - benign paroxysmal positional, Vestibular neuronitis
P Psychological	Attention deficit hyperactivity disorder, Autism in adults, Autism in children, Benzodiazepine and z-drug withdrawal, Bipolar disorder, Delirium, Depression, Depression - antenatal and postnatal, Depression in children, Dyspepsia - proven functional, Eating disorders, Generalized anxiety disorder, Insomnia, Irritable bowel syndrome, Learning disabilities, Mental health in students, Obsessive-compulsive disorder, Post-traumatic stress disorder, Postnatal and antenatal depression, Problem drinking - alcohol, Psychosis and schizophrenia, Schizophrenia and psychosis, Self-harm
R Respiratory	Allergic rhinitis, Asthma, Breathlessness, Bronchiectasis, Chest infections - adult, Chest pain, Chronic obstructive pulmonary disease, Common cold, Coronavirus - COVID 19, Corticosteroids - inhaled, Cough, Cough - acute with chest signs in children, Croup, Epistaxis (nosebleeds), Immunizations - pneumococcal, Immunizations - seasonal influenza, Influenza - seasonal, Influenza (seasonal) - immunizations, Lung and pleural cancers - recognition and referral, Nosebleeds (epistaxis), Obstructive sleep apnoea syndrome, Palliative care - cough, Palliative care - dyspnoea, Palliative care - secretions, Pneumococcal immunizations, Pulmonary embolism, Sore throat - acute, Tuberculosis, Whooping cough
S Skin	Acne vulgaris, Alopecia areata, Alopecia, androgenetic - female, Alopecia, androgenetic - male, Animal and human bites, Bites - human and animal, Bites and stings - insect, Boils, carbuncles, and staphylococcal carriage, Burns and scalds, Candida - skin, Carbuncles, boils and staphylococcal carriage, Cellulitis - acute, Chickenpox, Chilblains, Corticosteroids - topical (skin), nose, and eyes, Dermatitis - contact, Eczema - atopic, Fungal nail infection, Fungal skin infection - body and groin, Fungal skin infection - foot, Fungal skin infection - scalp, Hand foot and mouth disease, Head lice, Herpetic whitlow - and staphylococcal, Hirsutism, human and animal bites, Hyperhidrosis, Impetigo, Insect bites and stings, Itch - widespread, Itch in pregnancy, Lacerations, Melanoma and pigmented lesions, Molluscum contagiosum, MRSA in primary care, Nappy rash, Neck lump, Palliative care - malignant skin ulcer, Paronychia - acute, Pigmented lesions and melanoma, Pityriasis rosea, Pityriasis versicolor, Psoriasis, Rosacea, Scabies, Scalds and burns, Seborrhoeic dermatitis, Shingles, Skin cancers - recognition and referral, Staphylococcal carriage, boils and carbuncles, Staphylococcal whitlow - and herpetic, Urticaria, Venous eczema and lipodermatosclerosis, Veruccae and warts, Vitiligo, Warts - anogenital, Warts and verrucae, Whitlow (staphylococcal and herpetic)
T Metabolic, endocrine, nutritional	Addison's disease, Alcohol - problem drinking, Cholecystitis - acute, Cirrhosis, Diabetes - type 1, Diabetes - type 2, Diabetes type 1 - insulin therapy, Diabetes type 2 - insulin therapy, Food allergy, Gilbert's syndrome, Hepatitis A, Hepatitis B, Hepatitis C, Herpes simplex - genital, Hypercalcaemia, Hyperthyroidism, Hyponatraemia, Hypothyroidism, Insulin therapy in type 1 diabetes, Insulin therapy in type 2 diabetes, Jaundice in adults, Jaundice in the newborn, Neck

	lump, Non-alcoholic fatty liver disease (NAFLD), Obesity, Pancreatitis - acute, Pancreatitis - chronic, Type 1 diabetes, Type 1 diabetes - insulin therapy, Type 2 diabetes, Type 2 diabetes - insulin therapy, Vitamin D deficiency in adults, Vitamin D deficiency in children
U Urinary	Acute kidney injury, Bedwetting (enuresis), Chronic kidney disease, Colic - renal or ureteric (acute), Enuresis - bedwetting, Incontinence - urinary, in women, Kidney disease - chronic, Kidney injury - acute, LUTS in men, Nocturnal enuresis - bedwetting, Pyelonephritis - acute, Renal or ureteric colic - acute, Urinary incontinence in women, Urinary tract infection - children, Urinary tract infection (lower) - men, Urinary tract infection (lower) - women, Urological cancers - recognition and referral
W Pregnancy, family planning	Antenatal and postnatal depression, Antenatal care - uncomplicated pregnancy, Breastfeeding problems, Contraception - assessment, Contraception - barrier methods and spermicides, Contraception - combined hormonal methods, Contraception - emergency, Contraception - IUS/IUD, Contraception - natural family planning, Contraception - progestogen-only methods, Contraception - sterilization, Dyspepsia - pregnancy-associated, Ectopic pregnancy, Hypertension in pregnancy, Infertility, Itch in pregnancy, Menopause, Miscarriage, Nausea/vomiting in pregnancy, Postnatal and antenatal depression, Pre-conception - advice and management, Pregnancy (uncomplicated) - antenatal care
X Female genital	Amenorrhoea, Bacterial vaginosis, Breast abscess and mastitis, Breast cancer - managing FH, Breast cancer - recognition and referral, Breast pain - cyclical, Breast screening, Candida - female genital, Cervical cancer and HPV, Cervical screening, Chlamydia - uncomplicated genital, Dysmenorrhoea, Endometriosis, Fibroids, Gonorrhoea, Gynaecological cancers - recognition and referral, Herpes simplex - genital, HPV and cervical cancer, Mastitis and breast abscess, Menopause, Menorrhagia, Ovarian cancer, Pelvic inflammatory disease, Polycystic ovary syndrome, Premenstrual syndrome, Pruritus vulvae, Pubic lice, Syphilis, Trichomoniasis, Vaginal discharge, Warts - anogenital
Y Male genital	Balanitis, Chlamydia - uncomplicated genital, Erectile dysfunction, Gonorrhoea, Haematospermia, Herpes simplex - genital, Prostate cancer, Prostatitis - acute, Prostatitis - chronic, Pubic lice, Scrotal pain and swelling, Syphilis, Trichomoniasis, Undescended testes, Urethritis - male, Varicocele, Warts - anogenital
Z Social	Child maltreatment - recognition and management, Conduct disorders in children and young people, Domestic violence and abuse, Smoking cessation, Support for adult carers

Appendix C: Custom Stopword Dictionary

The custom stopword list was written by our clinical author.

also	difference	may	past	taken	want
another	effect	mind	people	thinking	water
around	fine	month	question	thought	way
back	four	months	quite	three	week
bad	good	morning	rather	time	weeks
better	help	need	ray	topic	weight
care	high	never	result	try	well
cause	important	new	right	trying	worried
come	information	night	ring	two	worry
cks	left	normal	side	use	year
day	life	often	six	using	
days	like	one	sometimes	usually	

Appendix D: Classifier Hyperparameters

All hyperparameter tuning was performed using cross validation on the OIAM training split. The classifiers were trained using conventional supervised learning with OIAM transcripts as training examples.

The SVM classifier uses a radial basis function kernel and L2 regularisation. We compared regularisation strengths of C=1, 2, 10, and *100,* finding the best performance with *C=2*, in both multiclass and multilabel modes, which we then use to produce all results shown in the main paper.

Our naïve Bayes (NB) classifier uses multinomial distributions with a smoothing parameter of $\alpha=0.001$ for both multiclass and multilabel modes. We tested values of *0.001, 0.01, 0.1, 1, 10,* and *100.* For multiclass NB, we found a small improvement when the class probabilities (priors) were fixed to a uniform distribution, and so also use this setting to produce the results in the main paper.

For the BERT classifiers, the maximum number of epochs was set to 15 and batch size to 8. Using the AdamW optimizer, we tuned the learning rate and weight decay rate, finding $5e^{-5}$ for the learning rate and $1e^{-4}$ for weight decay for all the PubMedBERT classifier variants tested here. Documents above 512 tokens were broken into chunks containing whole sentences with up to 490 tokens per chunk. Initially, we split 15% of the training data off as a validation set to perform early stopping. However, since this reduces the small training dataset further, we found that this harmed performance. Therefore, we perform early stopping only if the training set performance has converged. This is a likely contributor to overfitting, which motivates the creation of a larger training dataset in future work.
